# Clinical and Hematological Parameters and Their Correlation With the Severity of Vitamin B12 Deficiency: A Hospital-Based Study

**DOI:** 10.7759/cureus.91907

**Published:** 2025-09-09

**Authors:** Shilpy, Minakshi Mishra, Sapna Sandeep Saha, Ashutosh Kumar

**Affiliations:** 1 Pathology, Sir Sunderlal Hospital, Banaras Hindu University, Varanasi, IND; 2 Pathology, Tata Main Hospital, Jamshedpur, IND; 3 Cardiology, Heart Centre, Seva Sadan, Ranchi, IND

**Keywords:** anemia, clinico-hematological parameters, hypersegmented neutrophils, macrocytosis, vitamin b12 deficiency

## Abstract

Background

Vitamin B12 is an essential micronutrient for optimal hematopoietic and neurocognitive function. Vitamin B12 deficiency is associated with a wide spectrum of diseases affecting various systems of the body. This study aimed to investigate the clinico-hematological parameters in patients with vitamin B12 deficiency and analyze their correlation with severity.

Methodology

This hospital-based, prospective observational study was conducted at the Department of Pathology, Tata Main Hospital, Jamshedpur, over 12 months (June 2022-May 2023). Vitamin B12 testing was not routinely performed for all OPD and IPD patients but was ordered based on clinical discretion; therefore, patients with serum vitamin B12 <180 pg/mL were included, regardless of anemia status. During the study period, a total of 420 adult patients were classified as deficient (100-179 pg/mL) or severely deficient (<100 pg/mL). Sociodemographic, clinical, and hematological parameters were collected and analyzed for correlation with the severity of vitamin B12 deficiency.

Results

Among 9,596 patients tested for vitamin B12, 2,193 had levels <180 pg/mL, giving a prevalence of 22.9% among tested individuals. Out of 420 patients who met our eligibility criteria, 268 participants (63.8%) were classified as vitamin B12 deficient, while 152 participants (36.2%) were severely deficient. The most common clinical features were pallor (236, 56.2%), fatigue (160, 38.10%), anorexia (156, 37.14%), and tingling sensations (135, 32.14%). Anemia was present in 315 participants (75%), with higher rates of moderate and severe anemia in the severely deficient group (*P *< 0.001). Contrary to expectations, normocytic normochromic blood picture (176, 41.90%) and normocytic normochromic anemia (126, 30.00%) were more common than macrocytic anemia (9, 2.14%). The proportion of patients with irritability, numbness, and glossitis was significantly higher in severely deficient patients (*P *< 0.05). Mean corpuscular volume (MCV), mean corpuscular hemoglobin (MCH), mean corpuscular hemoglobin concentration (MCHC), and red cell distribution width (RDW) values were significantly higher in the severely deficient group (*P *< 0.05). Bicytopenia and pancytopenia were observed in 11 patients (2.6%) and 10 patients (2.38%), respectively, and occurred exclusively in those with severe vitamin B12 deficiency.

Conclusions

Vitamin B12 deficiency has a significant prevalence with distinct clinico-hematological correlations to severity. The correlation between the severity of deficiency and clinical manifestations, particularly neurological symptoms and the degree of anemia, underscores the importance of early detection and treatment. The predominance of normocytic rather than macrocytic anemia challenges traditional diagnostic paradigms and suggests clinicians should not rely solely on macrocytosis when suspecting vitamin B12 deficiency.

## Introduction

Vitamin B12 (cobalamin) represents an essential water-soluble micronutrient primarily obtained from animal-derived foods. This critical vitamin functions as a cofactor in DNA synthesis and plays a fundamental role in erythropoiesis, myelination, and cognitive function [1,2]. The human body maintains vitamin B12 stores that can last up to five years, which explains why deficiency manifestations develop gradually over extended periods [3]. Deficiency of vitamin B12 represents a significant public health concern worldwide, with prevalence varying considerably across different regions and populations. In developed countries, the prevalence ranges from 3% to 5% in the general population but increases to 10-15% among elderly individuals [4,5]. In developing nations, particularly those with predominantly vegetarian populations like India, the prevalence can exceed 70% in certain demographic groups [6].

The etiology of vitamin B12 deficiency encompasses multiple factors, including inadequate dietary intake (particularly among vegetarians), malabsorption syndromes, gastrointestinal disorders, certain medications (including metformin and proton pump inhibitors), and pernicious anemia [7,8]. The classic understanding of vitamin B12 deficiency associates it primarily with megaloblastic anemia, characterized by macrocytosis and hypersegmented neutrophils. However, the clinical and hematological manifestations can be heterogeneous and may deviate from this classical picture [9]. Clinical presentations of vitamin B12 deficiency span multiple organ systems, mainly involving hematological, neurological, and gastrointestinal manifestations [10]. However, many patients may remain asymptomatic or present with vague, non-specific complaints that can delay diagnosis [11]. Since such a deficiency is reversible, early initiation of treatment will reverse even the severe manifestations, and early preventive measures can be taken.

Despite its clinical significance, there is limited research exploring the correlation between the severity of vitamin B12 deficiency and its multisystem manifestations, particularly in the Indian population [12,13]. Therefore, this study was undertaken to investigate the clinical and hematological parameters in patients with vitamin B12 deficiency and analyze their correlation with severity at a tertiary care hospital in Eastern India.

## Materials and methods

This hospital-based, prospective observational study was conducted in the Department of Pathology, Tata Main Hospital, Jamshedpur, from June 2022 to May 2023. The institutional ethics committee approved the study protocol prior to participant recruitment.

Study participants

We included adult patients (≥18 years) attending either outpatient or inpatient departments who demonstrated serum vitamin B12 levels below 180 pg/mL on laboratory investigation. It needs to be emphasized that in the study hospital, vitamin B12 testing was ordered based on clinical discretion, not routinely performed for all Outpatient Department (OPD) and Inpatient Department (IPD) patients. This reflects a selective screening approach. Besides, we did not consider anemia as an inclusion criterion to avoid selection bias and allow analysis of hematological parameters across the spectrum of vitamin B12 deficiency, including non-anemic individuals. Exclusion criteria encompassed refusal to provide informed consent, recent blood transfusion (within three months), current vitamin B12 supplementation (within six months), pregnancy, critical illness requiring intensive care admission, known malignancy, and HIV/AIDS.

Sample size determination

Using the World Health Organization standard formula for prevalence studies and referencing previous research by Singla et al. [13] indicating a 47% prevalence of vitamin B12 deficiency in North India, we calculated a required sample size of 383 participants (95% confidence level, 5% margin of error). Accounting for a 10% potential dropout rate, we established a target of 420 participants. A non-probability consecutive sampling was done till the minimum sample size was met.

Data collection

After obtaining informed written consent, we collected comprehensive data using a structured proforma. Demographic information and laboratory parameters were retrieved from the hospital's Health Management Information System (HMIS), while clinical details were obtained through direct patient interviews. Neuropsychiatric symptoms like depression and memory disturbance were self-reported by patients; no formal screening tools (e.g., Patient Health Questionnaire-9 (PHQ-9) or Montreal Cognitive Assessment (MoCA)) were used. Blood samples were collected in ethylenediaminetetraacetic acid (EDTA) and plain vials for complete blood count and biochemical analyses, respectively.

We documented the data pertaining to *demographic characteristics *(age, gender, dietary pattern, and addiction), *medical history *(such as pre-existing malabsorption syndromes, gastrointestinal surgeries, medication use particularly metformin), and *clinical manifestations* (fatigue, anorexia, pallor, neuropsychiatric symptoms, and mucocutaneous signs) were noted from their medical records. *Hematological parameters *included were complete blood count including hemoglobin, red blood cell indices (mean corpuscular volume (MCV), mean corpuscular hemoglobin (MCH), mean corpuscular hemoglobin concentration (MCHC), and red cell distribution width (RDW)), white blood cell count, platelet count, and peripheral blood smear findings.

Laboratory methods

Serum vitamin B12 testing was performed in-house using the Beckman Coulter DXi 800 (Beckman Coulter, Inc., Brea, CA) and was quantified using chemiluminescence immunoassay (CLIA) with a measurement range of 50-1500 pg/mL [14]. Complete blood counts were performed using the DXH800 Beckman Coulter analyzer. Peripheral blood smears were prepared, stained with Leishman’s stain, and examined by experienced pathologists from the Department of Pathology; however, interobserver agreement was not evaluated. Two distinct blood pictures were observed, namely, *normocytic normochromic blood picture* and *normocytic normochromic anemia*. The first group had normal morphology and hemoglobin, while the second group had normal morphology but reduced hemoglobin. Anemia was classified according to WHO criteria: mild anemia = Hb 11-11.9 g/dL (females), 11-12.9 g/dL (males); moderate = 8-10.9 g/dL; severe = <8 g/dL [15]. Based on serum vitamin B12 levels, participants were categorized into two groups, namely, the deficient group (vitamin B12 level between 100 and 179 pg/mL) and the severely deficient group (vitamin B12 level <100 pg/mL) [16]. Cutoff values were based on hospital laboratory reference ranges.

Statistical analysis

Data analysis was conducted using SPSS for Windows, Version 23.0 (IBM Corp., Armonk, NY). Continuous variables were expressed as mean ± standard deviation, while categorical variables were presented as frequencies and percentages. Normality of data was assessed using the Shapiro-Wilk test. Comparisons between the deficient and severely deficient groups were performed using independent t-tests for normally distributed continuous variables, Mann-Whitney U tests for non-parametric data, and Chi-square or Fisher's exact (if expected count in any of the cell is less than 5) tests for categorical variables. Correlation analyses were conducted using Pearson's or Spearman's correlation coefficients as appropriate. A *P*-value < 0.05 was considered statistically significant.

## Results

Data from the HMIS of the hospital was accessed, which provided the pathological records for 11,031 patients during the study period, out of which blood investigation for vitamin B12 was conducted for 9,596 patients, and 2,193 demonstrated levels below 180 pg/mL, representing a prevalence of 22.9%. From these eligible patients, we enrolled 420 participants who met our eligibility criteria. Among the study cohort, 268 participants (63.8%) were classified as vitamin B12 deficient (100-179 pg/mL) with a mean level of 138.17 ± 23.84 pg/mL, while 152 participants (36.2%) were severely deficient (<100 pg/mL), with a mean level of 72.63 ± 17.02 pg/mL. The overall mean vitamin B12 level was 114.45 ± 38.22 pg/mL.

Table 1 presents the baseline characteristics of the study participants. The mean age of participants was 46.92 ± 17.62 years (range: 18-86 years). Gender distribution showed a slight female predominance (*n* = 222, 52.86%), with a male-to-female ratio of 0.89:1. In terms of dietary patterns, 161 (38.33%) followed vegetarian diets, while 198 (61.67%) consumed non-vegetarian diets. Twenty-one (3.81%) were alcoholics, and 32 (7.62%) were smokers. Medical history revealed that 5 participants (1.19%) had known malabsorption syndromes and 4 (0.95%) had undergone gastrointestinal surgeries. Notably, 45 patients (10.71%) reported long-term metformin use, which was significantly more common in the severely deficient group (*n* = 30, 19.74%, vs. *n* = 15, 5.60%; *P *< 0.001).

**Table 1:** Baseline characteristics of the study participants (n = 420). *Mean ± SD age: 46.92 ± 17.62 years; median (IQR): 45 (32-57) years. SD, standard deviation; IQR, interquartile range

Parameters		No. of patients	Percentage
Age (years)*	≤20	17	4.05%
	21-30	78	18.57%
	31-40	76	18.09%
	41-50	67	15.95%
	51-60	84	20.00%
	61-70	48	11.43%
	71-80	43	10.24%
	81-90	07	1.67%
Gender	Male	198	47.14%
	Female	222	52.86%
Diet	Vegetarian	161	38.33%
	Non-vegetarian	259	61.67%
Addiction	Alcoholic	21	5.00%
	Smoker	32	7.62%
Medical history	Known malabsorption syndrome	05	1.19%
	Gastrointestinal surgery	04	0.95%
	Metformin	45	10.71%

Pallor represented the most frequent clinical finding, observed in 236 participants (56.19%), followed by fatigue (*n* = 160, 38.10%), anorexia (n = 156, 37.14%), and tingling sensations (*n *= 135, 32.14%). Less common manifestations included glossitis (*n* = 53, 12.62%), irritability (*n* = 48, 11.43%), memory disturbances (*n* = 38, 9.05%), depression (*n* = 36, 8.57%), numbness (*n* = 33, 7.86%), skin hyperpigmentation (*n* = 33, 7.86%), cheilosis (*n* = 20, 4.76%), and ataxia (*n* = 14, 3.33%).

Comparison between severity groups revealed significant differences in several clinical features (Table 2). Patients with severe deficiency demonstrated higher rates of irritability (*P *= 0.034), numbness (*P *< 0.001), and glossitis (*P *< 0.001) compared to those with moderate deficiency. Interestingly, anorexia was significantly more prevalent in the moderately deficient group (*P *< 0.001).

**Table 2:** Comparison of clinical symptoms with severity of vitamin B12 deficiency among study participants (n = 420). ^#^Based on the Chi-square test. *P < 0.05 was considered as statistically significant.

Clinical symptoms	Severely deficient (*n* = 152)		Deficient (*n* = 268)		Chi-square statistic	*P*-value^#^
	No.	Percentage	No.	Percentage		
Fatigue (*n* = 160)	58	38.16%	102	38.06%	0.001	0.983
Anorexia (*n* = 156)	35	23.03%	121	45.15%	19.396	<0.001*
Pallor (*n* = 236)	91	59.87%	145	54.10%	1.085	0.252
Irritability (*n* = 48)	24	15.79%	24	8.96%	3.826	0.034*
Memory disturbance (*n* = 38)	17	11.18%	21	7.84%	0.946	0.252
Depression (*n* = 36)	13	8.55%	23	8.58%	0.001	0.996
Tingling sensation (*n* = 135)	39	25.66%	96	35.82%	4.139	0.032*
Numbness (*n* = 33)	23	15.13%	10	3.73%	15.872	<0.001*
Ataxia (*n* = 14)	08	5.26%	06	2.24%	1.895	0.097
Skin hyperpigmentation (*n* = 33)	15	9.87%	18	6.72%	0.931	0.249
Cheilosis (*n* = 20)	10	6.58%	10	3.73%	1.163	0.188
Glossitis (*n* = 53)	38	25%	15	5.60%	31.379	<0.001*

Out of 420 participants, anemia was present in 315 participants (75%) overall, with differing distributions according to deficiency severity, while 105 (25%) had normal hemoglobin levels despite being vitamin B12 deficient. In the severely deficient group, 42 (33.87%) had mild anemia, 52 (41.94%) had moderate anemia, and 30 (24.19%) had severe anemia. In contrast, the deficient group showed higher proportions of mild anemia (*n *= 98, 51.31%), similar rates of moderate anemia (*n *= 73, 38.22%), and lower rates of severe anemia (*n* = 20, 10.47%). This difference in anemia severity distribution was statistically significant (*P *< 0.001). The mean hemoglobin levels were significantly lower in severely deficient patients (9.61 ± 2.37 g/dL) compared to deficient patients (10.61 ± 1.77 g/dL, *P *< 0.001) (Table 3). A positive correlation was observed between vitamin B12 levels and hemoglobin (*r*= 0.253, *P *< 0.001).

**Table 3:** Comparison of severity of anemia with severity of vitamin B12 deficiency among study participants (n = 315). ^#^Based on the Chi-square test (for severity of anemia) and Mann-Whitney U test (for mean Hb level). *P < 0.05 was considered as statistically significant.

Severity of anemia	Severely deficient (*n *= 124)		Deficient (*n *= 191)		U-statistic or Chi-square statistic	*P*-value^#^
	No. of patients	Percentage	No. of patients	Percentage		
Mild anemia (*n *= 140)	42	33.87%	98	51.31%	14.325	<0.001*
Moderate anemia (*n *= 125)	52	41.94%	73	38.22%		
Severe anemia (*n *= 50)	30	24.19%	20	10.47%		
Mean ± SD hemoglobin	9.61 ± 2.37 gm/dL		10.61 ± 1.77 gm/dL		-4.026	<0.001*

Peripheral blood smear examination revealed predominance of normocytic normochromic blood picture (*n* = 176, 41.90%) and normocytic normochromic anemia (*n *= 126, 30%), while dimorphic anemia was observed in 94 cases (22.38%). Notably, the classic finding of macrocytic anemia was identified in only 9 participants (2.14%), though its prevalence was significantly higher in the severely deficient group (*P *= 0.008). Similarly, hypersegmented neutrophils were observed in merely 12 cases (2.86%) but were significantly more common in severe deficiency (*P *= 0.004) (Table 4).

**Table 4:** Comparison of presence of peripheral smear findings with severity of vitamin B12 deficiency among study participants (n = 420). ^#^Based on Chi-square test. ^a^Normocytic normochromic blood picture includes patients with hemoglobin in normal range and normal morphology on peripheral smear. ^*^P < 0.05 was considered as statistically significant.

Peripheral smear findings	Severely deficient (*n *= 152)		Deficient (*n *= 268)		Chi-square statistic	*P*-value^#^
	No. of patients	Percentage	No. of patients	Percentage		
Normocytic normochromic blood picture^a^ (*n *= 176)	59	38.82%	117	43.66%	0.934	0.334
Normocytic normochromic anemia (*n *= 126)	40	26.32%	86	32.09%	1.540	0.215
Dimorphic anemia (*n *= 94)	40	26.32%	54	20.15%	2.213	0.145
Macrocytic Anaemia (*n *= 9)	07	4.61%	02	0.75%	6.999	0.008*
Microcytic anemia (*n *= 15)	06	3.95%	09	3.36%	0.098	0.754
Hypersegmented neutrophils (*n *= 12)	09	5.92%	03	1.12%	8.057	0.004*

Red cell indices analysis showed significantly higher mean MCV (92.93 ± 14.42 fL vs. 88.85 ± 8.86 fL, *P *< 0.001), MCH (30.95 ± 20.45 pg vs. 28.15 ± 3.88 pg, *P *= 0.030), and RDW (16.71 ± 4.37% vs. 15.22 ± 6.78%, *P *= 0.006) in severely deficient patients compared to deficient patients (Table 5). A significant negative correlation existed between vitamin B12 levels and MCV (*r *= -0.209, *P *< 0.001).

**Table 5:** Comparison of mean value of hematological parameters with severity of vitamin B12 deficiency among study participants (n = 420). Values are presented as mean ± SD or *n* (%) ^#^Based on Chi-square test (for thrombocytopenia) and Mann-Whitney U test (for other hematological parameters). ^*^P < 0.05 was considered as statistically significant. SD, standard deviation

Hematological parameters	Vitamin B12 level		U-statistic or Chi-square statistic	*P*-value^#^
	Severely deficient	Deficient		
Hematocrit (HCT)	36.81 ± 6.72	37.30 ± 7.44	0.671	0.502
Mean corpuscular volume (MCV)	92.93 ± 14.42	88.85 ± 8.86	3.590	<0.001*
Mean corpuscular hemoglobin (MCH)	30.95 ± 20.45	28.15 ± 3.88	2.175	0.030*
Mean corpuscular hemoglobin concentration (MCHC)	31.46 ± 1.38	31.50 ± 1.35	0.289	0.772
Mean platelet volume (MPV)	11.70 ± 6.54	11.79 ± 8.31	0.115	0.908
Red cell distribution width (RDW)	16.71 ± 4.37	15.22 ± 6.78	2.734	0.006*
Thrombocytopenia (<1.5 x10^5^/μL)	71 (46.71%)	109 (40.67%)	0.259	0.867

Bicytopenia was noted in 11 patients (2.6%) and pancytopenia in 10 patients (2.38%), both exclusively observed among those with severe vitamin B12 deficiency. The mean vitamin B12 levels in these subgroups were 97.64 ± 49.70 and 90.90 ± 46.79 pg/mL, respectively.

## Discussion

This prospective study provides comprehensive insights into the clinico-hematological profile of vitamin B12 deficiency and its correlation with severity in an Eastern Indian population. Our finding of a 22.9% prevalence of vitamin B12 deficiency among hospital attendees undergoing the said laboratory investigation underscores the significant burden of this nutritional deficiency, which is somewhat lower than that reported by other studies across the country [13,16]. The study by Singla et al. [13] reported a 47% prevalence of vitamin B12 deficiency among adults in a city in North India. Another study by Samson et al. [17] reported vitamin B12 deficiency among adult patients in a tertiary hospital in South India to be 58.8% (26.1% were in deficient levels and 32.8% were in the severely deficient group). Differences in zonal prevalence of vitamin B12 deficiency can be explained by varying cutoff values of vitamin B12 taken up by individual studies to report deficiency. While Singla et al. [13] considered levels <200 pg/mL as deficient in vitamin B12, Samson et al. [17] used the cutoff value of 170 pg/mL to define their patients as deficient in vitamin B12, as opposed to the present study, which used the laboratory reference cutoff of 180 pg/mL at our hospital. Also, socioeconomic differences, mainly dietary differences across different states of the country, are speculated to contribute to the differences in the prevalence of B12 deficiency.

Further, it must be emphasized that all these studies were conducted in hospital settings, where patients seek medical attention for specific complaints that may involve certain systems and ultimately influence their vitamin B12 status. It is presumed that the prevalence figure observed in our study is an underestimation of the real scenario. Since the signs and symptoms of vitamin B12 deficiency are non-specific, the determination of the deficiency status among our study participants is mostly an incidental finding. We can assume that patients with vitamin B12 deficiency attending the hospital represent only the tip of the iceberg, and community-based studies across different parts of the country would reveal the true burden of vitamin B12 deficiency and the need to address the issue with supplementation.

The demographic profile in our study revealed no significant association between age, gender, or dietary pattern and the severity of vitamin B12 deficiency. The lack of correlation between a vegetarian diet and deficiency severity contrasts with traditional understanding [18,19] but aligns with emerging evidence suggesting that inadequate B12 intake occurs across dietary patterns [20]. Even non-vegetarians may have insufficient intake if animal food consumption is limited in quantity or frequency. This finding has important public health implications, suggesting that awareness regarding vitamin B12 sources and requirements should target broader populations rather than focusing exclusively on vegetarians.

The significant association between metformin use and severe vitamin B12 deficiency observed in our study corroborates existing literature on metformin-induced malabsorption of vitamin B12 [21-23]. This finding emphasizes the importance of routine monitoring of vitamin B12 status in patients on long-term metformin therapy, as recommended by several clinical practice guidelines [24].

The clinical profile analysis revealed important correlations between deficiency severity and symptom manifestation. The significantly higher prevalence of neurological symptoms (irritability, numbness) and mucosal changes (glossitis) in severely deficient patients highlights the multisystem impact of profound deficiency. These findings align with a previous report by Kumar N. that neurological manifestations can be more prominent in severe deficiency [25], suggesting that such symptoms, particularly when occurring in combination, should prompt consideration of severe vitamin B12 deficiency in clinical practice [26].

Perhaps the most striking finding in our study concerns the hematological profile. We noted that one-fourth of the patients demonstrated normal hemoglobin levels despite being deficient in vitamin B12. This could be attributed to early-stage deficiency, individual variation in hematological response, or coexisting factors such as iron sufficiency, mitigating anemia development. Moreover, contrary to the classical teaching that associates vitamin B12 deficiency with macrocytic anemia, we found that normocytic blood pictures and anemia predominated, with macrocytic anemia present in only 2.14% of participants. This observation challenges conventional diagnostic paradigms and may explain why vitamin B12 deficiency remains underdiagnosed when clinicians rely primarily on macrocytosis as a diagnostic clue. Several factors may contribute to this discrepancy. First, coexisting iron deficiency (confirmed in 32% of tested patients in our study) can mask macrocytosis by reducing MCV values. Second, inflammatory conditions, common in hospital populations, can suppress erythropoiesis and modify red cell indices. Third, early or moderate deficiency may not yet manifest the classical hematological picture that typically develops with prolonged severe deficiency. Nevertheless, our finding that MCV, MCH, and RDW values were significantly higher in severely deficient patients, even while remaining within normal ranges in many cases, suggests that trends in these parameters rather than absolute values may provide valuable diagnostic clues. Clinicians should consider vitamin B12 deficiency even in the absence of overt macrocytosis, particularly when other clinical features suggest the diagnosis.

In cobalamin deficiency, platelet production is only 10% of that expected from megakaryocyte mass, perhaps reflecting ineffective thrombopoiesis, and they too are functionally abnormal [17]. In the present study, the overall proportion of patients with thrombocytopenia was 42.9%, which accounted for 46.7% of patients with severe deficiency of vitamin B12 and 40.7% of patients with deficient vitamin B12. Mahajan et al. reported thrombocytopenia in 8% of their patients with vitamin B12 deficiency and/or folic acid deficiency [27]. The exclusive occurrence of bicytopenia and pancytopenia in severely deficient patients highlights the profound impact of severe vitamin B12 deficiency on hematopoiesis by affecting multiple hematopoietic lineages [28]. This finding suggests that vitamin B12 deficiency should be considered in the differential diagnosis of unexplained cytopenias, even when classical macrocytosis is absent [29].

A handful of patients who underwent repeat blood investigations during the study period reported being on routine vitamin B12 supplementation or having received injectable vitamin B12 (in cases of severe deficiency) at the discretion of their treating physician, and were found to have improvements in their vitamin B12 status.

Strengths and limitations of the study

Our study has several strengths, including its prospective design, substantial sample size, and comprehensive assessment of clinical and hematological parameters. However, certain limitations warrant consideration. First, other potential confounding variables that could explain certain symptoms, such as diabetic neuropathy, spinal degenerative changes, or underlying psychiatric and neurological conditions, were not controlled for. Second, we were unable to stratify the presenting complaints and associated comorbidities according to the departments from which patients were referred due to the large sample size and resource limitations. However, most participants were from the Departments of Internal Medicine and Neurology. Third, while dietary pattern was recorded, an objective nutritional assessment or detailed evaluation of the non-vegetarian group could not be performed due to the limited duration of patient interviews; only their routine dietary habits were documented. Fourth, reliance on self-reported neurological symptoms without validated tools may have introduced reporting bias. Fifth, inter-observer variability in peripheral smear reviews was not assessed, which may affect the consistency of morphological interpretations. Lastly, advanced biochemical markers such as serum methylmalonic acid or homocysteine were not measured, bone marrow examination was not performed in most patients, and follow-up data on post-supplementation response were limited.

## Conclusions

This study demonstrates that vitamin B12 deficiency affects a substantial proportion of patients attending tertiary care facilities in Eastern India, with important correlations between deficiency severity and clinico-hematological parameters. The higher prevalence of neurological symptoms and glossitis in severely deficient patients provides valuable diagnostic clues for identifying profound deficiency in clinical practice. Given the potentially irreversible neurological consequences of prolonged vitamin B12 deficiency and the relatively simple and effective treatment options available, early detection through comprehensive clinical and hematological evaluation is crucial. Future research should focus on developing more accessible and cost-effective diagnostic approaches for vitamin B12 deficiency, particularly in resource-limited settings, and evaluating the effectiveness of population-level interventions such as food fortification programs.
